# Accumulation mechanism of metabolite markers identified by machine learning between Qingyuan and Xiushui counties in *Polygonatum cyrtonema* Hua

**DOI:** 10.1186/s12870-024-04871-6

**Published:** 2024-03-06

**Authors:** Qiqi Gong, Jianfeng Yu, Zhicheng Guo, Ke Fu, Yi Xu, Hui Zou, Cong Li, Jinping Si, Shengguan Cai, Donghong Chen, Zhigang Han

**Affiliations:** 1 State Key Laboratory of Subtropical Silviculture, Zhejiang A&F University, Hangzhou, 311300 China; 2 School of Forestry and Biotechnology, Zhejiang A&F University, Hangzhou, 311300 China; 3 Shandong Marine Resource and Environment Research Institute, Shandong Provincial Key Laboratory of Restoration for Marine Ecology, Yantai, 264006, China; 4 Yipuyuan Huangjing Technology Co., Ltd, Xinhua, 417600 China; 5 College of Agriculture and Biotechnology, Zhejiang University, Hangzhou, 310030 China

**Keywords:** *Polygonatum cyrtonema* Hua, Phenylpropanoids, Machine learning, Metabolomics, Biosynthesis

## Abstract

*Polygonatum cyrtonema* Hua is a traditional Chinese medicinal plant acclaimed for its therapeutic potential in diabetes and various chronic diseases. Its rhizomes are the main functional parts rich in secondary metabolites, such as flavonoids and saponins. But their quality varies by region, posing challenges for industrial and medicinal application of *P. cyrtonema*. In this study, 482 metabolites were identified in *P. cyrtonema* rhizome from Qingyuan and Xiushui counties. Cluster analysis showed that samples between these two regions had distinct secondary metabolite profiles. Machine learning methods, specifically support vector machine-recursive feature elimination and random forest, were utilized to further identify metabolite markers including flavonoids, phenolic acids, and lignans. Comparative transcriptomics and weighted gene co-expression analysis were performed to uncover potential candidate genes including *CHI*, *UGT1*, and *PcOMT10/11/12/13* associated with these compounds. Functional assays using tobacco transient expression system revealed that *PcOMT10/11/12/13* indeed impacted metabolic fluxes of the phenylpropanoid pathway and phenylpropanoid-related metabolites such as chrysoeriol-6,8-di-C-glucoside, syringaresinol-4'-O-glucopyranosid, and 1-O-Sinapoyl-D-glucose. These findings identified metabolite markers between these two regions and provided valuable genetic insights for engineering the biosynthesis of these compounds.

**Supplementary Information:**

The online version contains supplementary material available at 10.1186/s12870-024-04871-6.

## Background

**Fig. 1:**
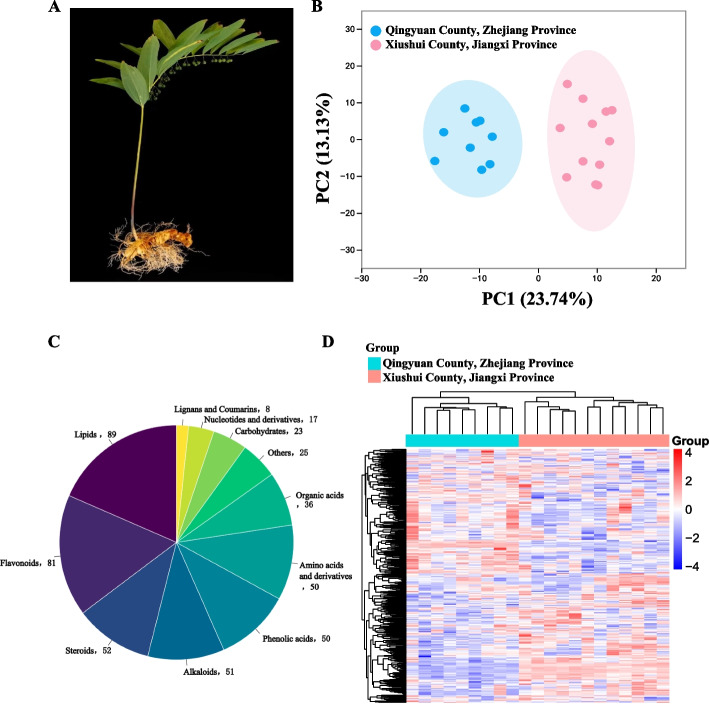
Metabolomic analysis of *P. cyrtonema* from two sites in Qingyuan and Xiushui Counties. **A** Diagram of *P. cyrtonema* plant. **B** PCA analysis of the two sites. **C** Component analysis of the identified metabolites from *P. cyrtonema*. **D** Complexheatmap of all metabolites identified by relative quantification

*Polygonatum cyrtonema* Hua is an ancient and traditional herbal plant in China (Fig. 1A). The rhizome of this plant, rich in a diverse array of secondary metabolites including triterpenoid saponins, steroids, and flavonoids, offers a myriad of health benefits [1]. These include the prevention of diabetes in obese individuals, enhancement of insulin secretion, improvement of insulin resistance, antibacterial properties, anti-tumor effects, anti-inflammatory properties and anti-aging benefits [2, 3]. *P. cyrtonema* primarily grown in forests in the southern region of China [4]. Studies have noted differences in the phytochemical compositions of *P. cyrtonema* from various regions [5, 6]. However, key metabolite markers of *P. cyrtonema* between regions is unclear, which will inevitably affect the development of related industrial and medicinal application. Hence, it is significant to detect specific metabolic indicators for differentiating the quality of *P. cyrtonema* grown in various regions.

Recently, machine learning methods have significantly enhanced the detection of metabolite markers in plant and human [7, 8]. The support vector machine-recursive feature elimination (SVM-RFE) is a common multivariate approach in machine learning that is extensively employed for selecting features and classifying high-throughput data [9]. Furthermore, the random forest (RF), known for its superior accuracy and interpretability, has gained prominence in predictive modeling for feature biomarkers [7, 10]. Consequently, machine learning has proven instrumental in efficiently identify metabolite markers from voluminous metabolomic datasets. Further elucidation of the underlying biosynthetic mechanisms of these signature metabolites across different regions is particularly crucial.

Accumulation of plant secondary metabolites are usually originated from the phenylpropanoid pathway, such as coumarins, lignans, flavonoids, phenolic acids, and lignins [11]. The phenylpropanoid pathway typically initiates in plants by synthesizing phenylalanine through the glycolysis and shikimic acid pathway [12–14]. Afterwards, phenylalanine ammonia lyase (PAL), cinnamate-4-hydroxylase (C4H), and 4-coumaryl-CoA ligase (4CL) catalyze the reaction in a sequential manner to generate cinnamic acid, hydroxycinnamic acid, and CoA-linked 4-coumarate, respectively [15]. These metabolites are directed into two principal downstream pathways: the biosynthesis of monophenols and flavonoids. Lignin production is primarily regulated by hydroxycinnamoyl transferase (HCT), which plays an essential role in controlling the subsequent monophenol metabolism [16]. This is followed by the catalytic activities of cinnamoyl-CoA reductase (CCR) and cinnamyl-alcohol dehydrogenase (CAD). Downstream flavonoid metabolism is believed to be controlled by the pivotal chalcone synthase (CHS). After CHS synthesizes the chalcone, it undergoes conversion to flavanone through the action of CHI. Subsequently, a sequence of enzymes including flavanone 3-hydroxylase (F3H), flavonol synthase (FLS), and flavonoid synthase (FNS) facilitate the generation of dihydroflavonols, quercetin, and apigenin, correspondingly [17]. Despite the extensive knowledge on the phenylpropanoid pathway genes in *A. thaliana*, the comprehension of biosynthesis of this way in *P. cyrtonema* remains largely elusive. Currently, application of weighted gene co-expression network analysis (WGCNA) has gained prominence for elucidating the genetic architecture underlying the biosynthesis of secondary metabolites, which integrates comprehensive transcriptomic and metabolomic data [18]. This method considers the association degree among genes by analyzing the pairwise correlations of gene expression profiles, which offers considerable advantages in the exploration of secondary metabolite biosynthesis [18, 19].

In this study, we used a widely targeted metabolome approach to identify 482 metabolites from 21 different wild genotypes of *P. cyrtonema* growing in Qingyuan and Xiushui counties. Subsequently, the RF and SVM-RFE machine learning techniques were employed to distinguish characteristic metabolites between Qingyuan and Xiushui counties. By comparative transcriptome and WGCNA analyses, candidate genes of these compounds were mined. The *PcOMT10/11/12/13* genes were characterized using tobacco transient transformation system. All these results revealed the biomarkers of metabolites in these two counties and their accumulation mechanism in *P. cyrtonema*.

## Materials and methods

### Plant materials and sampling

Twenty one wild genotypes of *P. cyrtonema* were collected from Qingyuan and Xiushui counties (Table S1) and transplanted to the plantation of Zhejiang A&F University. During the year 2017, the rhizomes of *P. cyrtonema* were disinfected using a 1% concentration of carbendazim for a duration of 60 min. Subsequently, they were washed with tap water and placed in a pot containing nutrient-rich soil. During the year 2020, three sets of *P. cyrtonema* rhizome samples with removal of fibrous roots were collected, rapidly frozen in liquid nitrogen and stored at -80 °C [20].

Identification and quantification of metabolites through widely targeted metabolome approach.

Metabolites were extracted, identified, and quantified following the method described by Han et al. (2023). In short, the rhizome samples were freeze-dried and then pulverized using a mixer (Retsch, Haan, Germany). The freeze-dried tissue (0.1 g) was dissolved in extraction buffer, and the mixture was incubated at 4 °C for 12 h. The extraction solution was centrifuged with 14,000 rpm for 8 min. Subsequently, the extracted samples were analyzed using an ultrahigh-performance liquid chromatography-electrospray ionization-tandem mass spectrometry (UPLC-ESI–MS/MS). Following UPLC, the effluent was connected in succession to an ESI-triple quadrupole linear ion trap (Q TRAP)-MS [20].

The triple quadrupole linear ion trap mass spectrometer (QTrap) was operated using Analyst 1.6 software (AB Sciex) to gather scans from the linear ion trap (LIT) and triple quadrupole (QQQ). Polypropylene glycol solutions at concentrations of 10 and 100 mol/L were used for instrument tuning and mass calibration for QQQ and LIT scans. QQQ scans were obtained using the multiple reaction monitoring (MRM) mode, with the collision gas (nitrogen) set at 5 psi. For each MRM transition, the DP and CE were optimized. The eluted metabolites were monitored at each time interval by monitoring a specific MRM transition (Fig. S1; Table S2) [20].

Simca-P software (version 13.0, Umetrics AB, Umea, Sweden) was used to perform orthogonal partial least squares discriminant analysis (OPLS-DA) and unsupervised principal component analysis (PCA) on the processed data. The R software was used to conduct hierarchical clustering analysis of metabolites across samples [21]. To identify differentially accumulated metabolites (DAMs), screening criteria such as log_2_(Fold Change) ≥ 1, *p-*value ≤ 0.05, or variable importance in the projection (VIP) ≥ 1 were applied. The metabolic data for these two wild genotypes (T118_21 and T26_14) were obtained from Han et al. (2023).

### Identification of signature metabolites accumulated in Qingyuan and Xiushui counties

According to Han et al. (2024), feature metabolites were identified by using the RF function of “caret” package. The selection was based on the significance of these metabolites in classification, considering the variations among the metabolome samples. To prevent sample size bias and overfitting, 100 replicas of the random forest model were trained and tested using a well-balanced ten-fold cross-validation approach. Using the “e1071” library in R, a support vector machine (SVM) model was built, and metabolites with an average rank below 30 were chosen as the featured metabolites. Next, we deduced the metabolites that overlapped between the two classifiable models [21].

### RNA sequencing and analysis

The MiniBEST Plant RNA Extraction Kit (TaKaRa) was used to extract total RNA. RNA concentration and quality were evaluated with the Agilent 2100 Bioanalyzer. A total of 45 RNA-seq libraries were created via NEB Next Ultra RNA Library Preparation Kit (NEB, E7530) and the NEB Next Multiplex Oligos (NEB, E7500). Next, paired-end sequencing was carried out using an Illumina Hiseq 2500 platform. The Trinity software was employed to compute the transcript abundance of genes in transcripts per million (TPM). Differentially expressed genes were identified with |log_2_ (fold change) |≥ 1 and *p-*value (Padj) < 0.05, as detected by DESeq [20]. The RNA-seq data included in this study were obtained from Han et al. (2023).

### Weighted Gene Co-expression Network Analysis (WGCNA)

The WGCNA package (version 1.6.6) in R software (version 3.4.4) was used to construct weighted gene co-expression networks and identify associated modules; *r* > 0.90 and *p* < 0.001 were defined as the criteria for identifying significant modules related to flavonoids, whereas *r* ≥ 0.80 and *p* ≤ 0.001 were established as the criteria for significant modules related to phenolic acids and lignans. Hub genes related to the compounds were considered to meet two criteria, including a single gene-trait correlation over 0.90, and an edge weight greater than 0.1. Gephi was used to display the network of co-expression genes [21]. The RNA-seq data included in this study were obtained from Han et al. (2023).

### Tobacco transient expression assay

*PcOMT10, PcOMT11, PcOMT12* and *PcOMT13* CDS were cloned into the modified plant expression vector pCAMBIA1380-35S::GFP and subsequently injected into *Agrobacterium tumefaciens* GV3101 [20]. All constructions were expressed transiently in *Nicotiana benthaniana* leaves. Three days after injection, positive transgenic leaves were identified using real-time quantitative PCR. Widely targeted metabolomics was used to identify phenylpropanoids in overexpressed transgenic tissues. In these trials, three biological replicates were employed.

### Extraction of RNA and real-time quantitative PCR (RT-qPCR)

Total RNA was extracted using the MiniBEST Plant RNA Extraction kit (TaKaRa, Japan). The cDNA was obtained by PrimerScript RT Enzyme Mix I kit (TaKaRa, Japan). RT-qPCR was analyzed by SYBR® Premix Ex Taq II (TaKaRa, Japan) and a CFX96 TouchTM Real-Time PCR System (BIO-RAD, USA). Melt-Curve analysis (60 °C—95 °C, 0.5 °C increment for 5 s per step) was used to test the amplicon specificity after the PCR was run as follows: 30 s at 95 °C for pre-denaturation, 40 cycles of 5 s at 95 °C for denaturation, and 30 s at 60 °C for annealing (Takara, Japan) [20]. The comparative Ct (Ct: cycle threshold) approach was applied for relative quantification. Each analysis required technical replicates and three independent biological replicates, respectively. Primers are listed in Table S3.

## Results

### Metabolite identification among 21 genotypes

To better understand fluctuating variations in bioactive substances among 21 different genotypes from Qingyuan and Xiushui counties, a total of 482 metabolites in *P. cyrtonema* rhizomes were successfully identified using UPLC-ESI–MS/MS (Table S4). The PCA analysis revealed a strong sample correlation within region, but a distinct separation between the two counties (Fig. 1B). These metabolites were then categorized into 11 distinct groups, which included lignans and coumarins (8 metabolites), lipids (89), nucleotides and derivatives (17), organic acids (36), steroids (52), phenolic acids (50), alkaloids (51), amino acids and derivatives (50), carbohydrates (23), flavonoids (81), and others (25) (Table S5; Fig. 1C and S2). Of those, phenylpropanoids accounted for 28.83% of the total metabolites. Clustering analysis of these compounds also demonstrated a noticeable differentiation between the two regions (Fig. 1D), suggesting variations in the accumulation of these metabolites under different cultivation conditions.

### Machine learning-based selection of the best metabolite markers

**Fig. 2:**
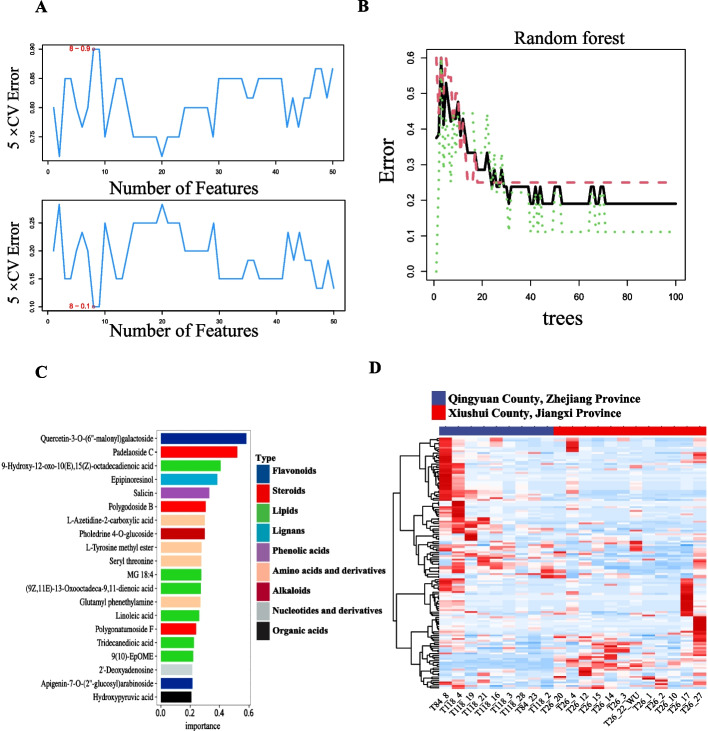
Machine learning methods identify characteristic metabolites. **A** SVM-RFE, a support vector machine—recursive feature elimination approach. **B** The number of decision trees and the relationship to the error rate. **C** Top 20 of feature metabolite results in the random forest method. **D** Complexheatmap of all phenylpropane metabolites in two sites

Machine learning methods were used to screen metabolite markers based on a total of 482 metabolites identified by widely targeted metabolome analysis. Based on the average rank and cv error, 30 feature metabolites were ultimately determined using SVF-RFE (Table S6; Fig. 2A). Furthermore, the top RF model, which relied on 44 trees, recognized 20 metabolites as feature indicators (Table S7; Fig. 2B and C). After the classification by RF, the metabolites that were considered most significant were quercetin-3-O-(6''-malonyl) galactoside, padelaoside C, 9-Hydroxy-12-oxo-10(E), 15(Z)-octadecadienoic acid, epipinoresinol, and salicin. Three overlapping metabolites identified by SVM-RFE and RF methods, including apigenin-7-O-(2''-glucosyl)arabinoside, L-Azetidine-2-carboxylic acid, seryl threonine were screened using Venn diagrams (Fig. S3). Finally, based on the significance of machine learning classification, three metabolites were selected as metabolite markers including quercetin-3-O-(6''-malonyl) galactoside, salicin and epipinoresinol, belonging to flavonoids, phenolic acids and lignans respectively. Furthermore, clustering analysis also revealed a distinct regional differentiation for the aforementioned three classes of coumpounds from the two regions (Fig. 2D), demonstrating that these compounds can effectively distinguish the quality of *P. cyrtonema* from the two locations.

### Mining and characterization of key candidate genes involved in flavonoid biosynthesis

**Fig. 3:**
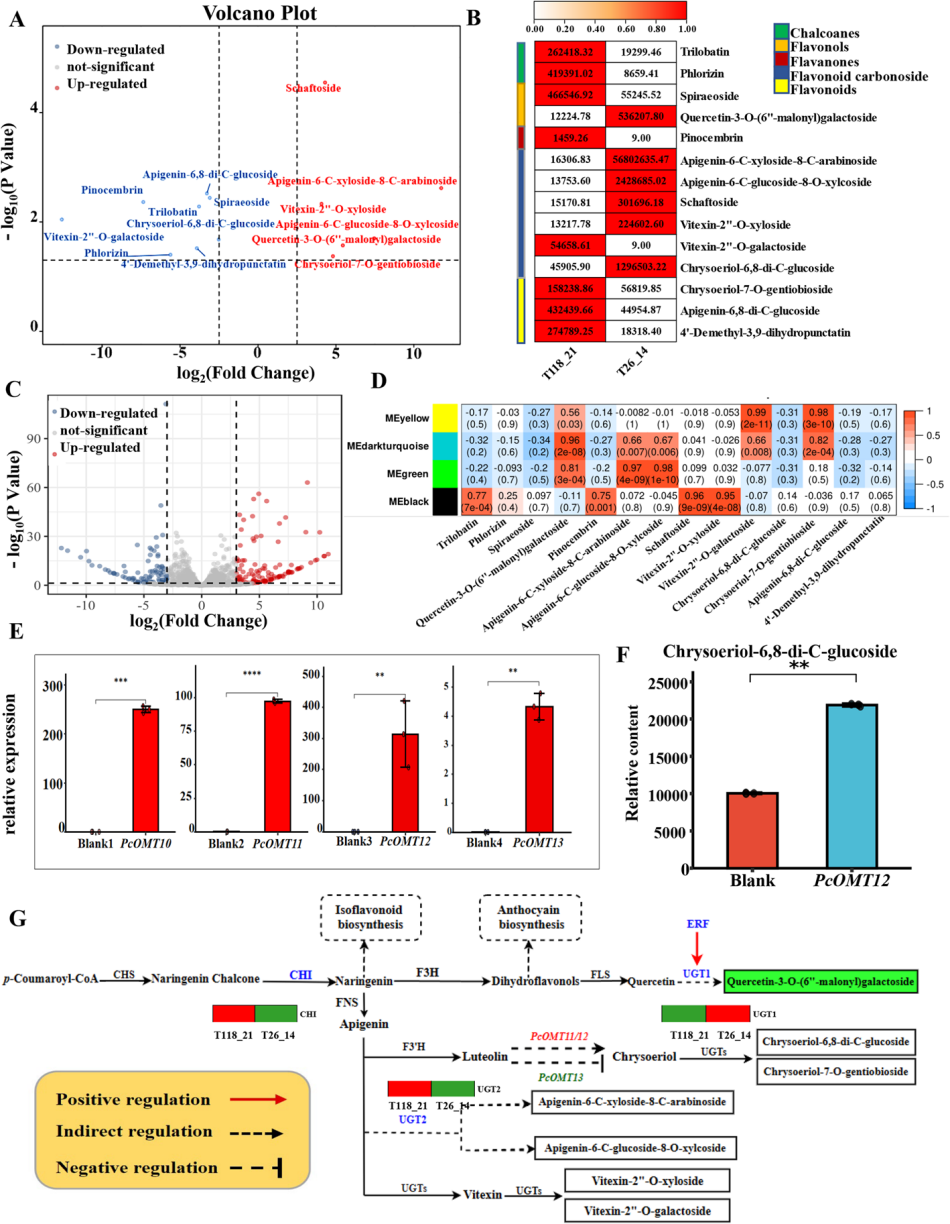
Mining of key candidate genes in flavonoid biosynthesis. **A** Volcano plot of flavonoids metabolites; **B** Relative content of flavonoids compounds. **C** Volcano map of differentially expressed genes (*p-*value < 0.05). **D** Correlation of flavonoids and modules. **E** qRT-PCR of *PcOMT10/11/12/13* in *N. benthaniana* leaves. **F** Changes in chrysoeriol-6,8-di-C-glucoside content after overexpression in *N. benthaniana* leaves. **G** Flavonoid biosynthesis pathway and expression of key genes in the *P. cyrtonema*. Green genes show that compound accumulation is inhibited, whereas red genes promote compound accumulation

Two genotypes T118_21 (Qingyuan County) and T26_14 (Xiushui County) were selected to conduct comparative analysis of flavonoid compounds, which showed that these metabolites had significantly different contents between the two genotypes (Fig. 3A and B).

Further, a comparative transcriptome analysis between the two genotypes was also conducted on the two genotypes. A total of 625 significantly differential expressed genes (DEGs) were identified (Fig. 3C). Four candidate genes related to flavonoids synthesis were selected fromt those DEGs (Fig. S4), and were named *PcOMT10, PcOMT11*, *PcOMT12* and *PcOMT13*, respectively, based on the phylogenetic analysis (Fig. S5; Table S8). WGCNA was also applied to identify significantly correlated metabolites-modules based on transcripts and 14 flavonoid metabolites (Fig. 3D). There was a strong correlation between apigenin-6-C-xyloside-8-C-arabinoside and apigenin-6-C-glucoside-8-O-xylcoside with the green module respectively, suggesting the similar accumulation mechanisms of these compounds involved in flavonoid biosynthesis. Further, hub genes in darkturquoise and green modules, including the flavonoid structural genes *CHI*, *UGT1* and *UGT2*, as well as an ERF gene that positively correlating with the *UGT1* gene, were identified.

To investigate their roles in the accumulation of flavonoid compounds, we constructed the corresponding plant expression vectors (Fig. S6) and performed transient transformation experiments in *N. benthaniana*. The qRT-PCR showed that the expression levels of *PcOMT11/12/13* were markedly upregulated compared with the control in *N. benthaniana* leaves (Fig. 3E). Notably, overexpression of *PcOMT12* resulted in a significant increase in the content of chrysoeriol-6,8-di-C-glucoside in *N. benthaniana* leaves (Fig. 3F). Furthermore, the overexpression of *PcOMT11* and *PcOMT13* led to a substantial increase and decrease, respectively, in the levels of chrysoeriol-7-O-gentiobioside. This indicates their respective roles in promoting and inhibiting accumulation (Fig. 3G).

### Mining and characterization of key candidate genes involved in phenolic acids and lignans biosynthesis

**Fig. 4:**
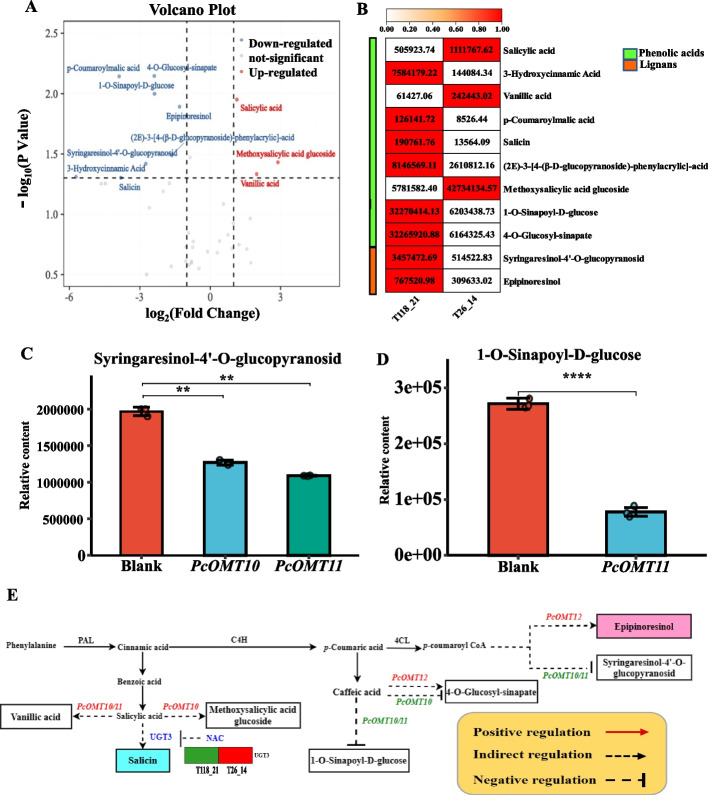
Mining of key candidate genes in the biosynthesis of phenolic acids and lignans. **A** Volcano plot of phenolic acids and lignans metabolites. **B** Heatmap of relative content of phenolic acids and lignans compounds. **C** Changes in syringaresinol-4'-O-glucopyranosid content after gene overexpression in *N. benthaniana* leaves. **D** Changes in 1-O-Sinapoyl-D-glucose content after gene overexpression in *N. benthaniana* leaves. **E** Phenolic acids and lignans biosynthesis pathway and expression of key genes in the *P. cyrtonema*. Green genes show that compound accumulation is inhibited, whereas red genes promote compound accumulation

Comparative analysis of content of phenolic acids and lignans two genotypes between T118_21 and T26_14 revealed there are 11 different metabolites, including three upregulated and eight downregulated metabolites (Fig. 4A and B). According to WGCNA analysis, there was a strong positive correlation between *p*-Coumaroylmalic acid and the blue module; salicin exhibited the highest correlation with the lightgreen module; epipinoresinol exhibited a strong correlation with the lightpink4 module (Fig. S7). Finally, we identified two candidate genes, including *UGT3* and *NAC* in the lightgreen module, and *NAC* might negatively regulate the *UGT3* gene. Based on comparative transcriptome analysis, three DEGs *PcOMT10*, *PcOMT11* and *PcOMT12* were identified. When *PcOMT10* and *PcOMT11* were overexpressed respectively in *N. benthaniana* leaves, the contents of both syringaresinol-4'-O-glucopyranosid (Fig. 4C) and 1-O-sinapoyl-D-glucose (Fig. 4D) were significantly decreased whereas the content of metabolite marker epipinoresinol increased with the overexpression of *PcOMT12* (Fig. 4E).

### Biosynthetic model of metabolite markers-related phenylpropanoid compounds in *P. cyrtonema*

**Fig. 5:**
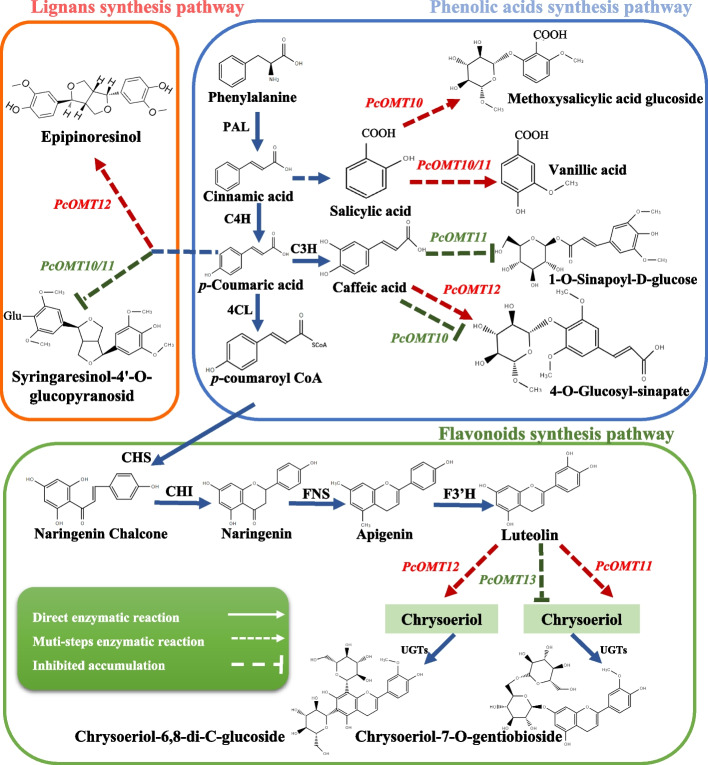
Analysis of the biosynthetic pathway of phenylpropane in *P. cyrtonema*. In the pathway diagram, red genes indicate promotion of compound accumulation, while green genes indicate inhibition of compound accumulation

Based on metabolite markers between the two regions and comparative transcriptome analysis of two representative genotypes, we constructed a biosynthetic model of important phenylpropanoids in *P. cyrtonema*. The manifestation of the four genes *PcOMT10/11/12/13* in *N. benthaniana*, were consistent with the pattern of compound accumulation in *P. cyrtonema* (Fig. 5). Surprisingly, *PcOMT12* has a promotional effect on the synthesis of several oxymethylated phenylpropane compounds, such as epipinoresinol, 4-O-glucosyl-sinapate and chrysoeriol-6,8-di-C-glucoside. Moreover, *PcOMT13* only somewhat inhibited the accumulation of chrysoeriol-7-O-gentiobioside content. The *PcOMT10/11* showed the same catalytic effect in the synthesis of syringaresinol-4'-O-glucopyranosid and vanillic acid.

## Discussion

*P. cyrtonema* is a Chinese traditional and classic dual-purpose plant for food and medicine with a number of biological properties, such as anti-aging, nourishing Yin, anti-inflammation, and immune regulation [2, 4]. In this study, large differences in rhizome metabolites of *P. cyrtonema* among different germplasms and regions have been investigated (Fig. S2). Identifying metabolite markers in non-model plants remains challenging. Traditional analytical methods may identify a larger number of metabolites; whereas with the use of machine learning methods it is able to focus on a few compounds with a larger weighting, which greatly narrows the scope [21]. Over the past few years, some researcher have begun using machine learning techniques for fruit flavor identification [7]. By machine learning techniques, ten metabolic biomarkers were identified to distinguish specific Chinese cherry accessions [22]. In this study, we detected significant metabolite markers including quercetin-3-O-(6"-malonyl) galactoside, salicin, and epipinoresinol linked to the distinctions between Qingyuan and Xiushui counties using machine learning methods. Each of these metabolites contributes significantly to the plant's growth and medicinal properties. Quercetin-3-O-(6"-malonyl) galactoside, for instance, is associated with the enzymatic browning of iceberg lettuce [23] and has been observed in higher concentrations in *Ribes nigrum* L. grown in northern regions compared to southern ones [24]. Salicin, commonly extracted from herbaceous plants, is used to alleviate pain in acute rheumatic conditions [25]. Additionally, forsythoside synthesis involves epipinoresinol, a prevalent compound in Forsythia [26].

Through WGCNA, our study identified four key pathway genes, including *CHI* and *UGT1/2/3*, as well as two regulatory genes, *ERF* and *NAC*, which specifically modulate *UGT1* and *UGT3*, respectively. CHI, catalyzing naringenin, serves as a pivotal intermediate in the flavonoid biosynthesis pathway [27]. Notably, we observed a positive correlation between CHI and vitexin-2"-O-galactoside in our analysis.

Plant glycosyltransferases, known for their extensive role in secondary metabolism, have been comprehensively characterized in various species, including Arabidopsis [28], cereals [29], and rice [30]. Intriguingly, despite a reduction in *UGT2* expression, we detected an increase in its associated metabolites, such as apigenin-6-C-xyloside-8-C-arabinoside and apigenin-6-C-glucoside-8-O-xylcoside. Furthermore, our findings imply that ERF may upregulate *UGT1*, promoting the accumulation of quercetin-3-O-(6"-malonyl) galactoside. Corroborating our results, Wan et al. (2023) reported that transient overexpression of *CsERF003* in citrus fruits markedly increased flavanones, flavonoids, and flavonols, alongside the upregulation of key genes involved in their biosynthesis, hinting at CsERF003's regulatory role in flavonoid biosynthesis via *UGT* expression modulation [31]. Our results also suggest *ERF* might enhance the expression of *UGT1* to facilitate the buildup of quercetin-3-O-(6"-malonyl) galactoside. Conversely, the NAC exhibited a negative correlation with *UGT3*, potentially enhancing salicin content. Previous studies have underscored NAC's vital role in plant stress responses, predominantly by modulating flavonoid synthesis [32]. In *A. thaliana*, the NAC transcription factor *ANAC078* regulates flavonoid biosynthesis in response to high light stress [33], while in Norway spruce, overexpression of *PaNAC03* led to diminished flavonol biosynthesis and aberrant embryo development [34]. These observations suggest that NAC may facilitate an increase in phenolic acid content while reducing flavonol levels.

The *O*-methyltransferase has garnered increasing attention due to its unexpectedly broad compatibility and selectivity towards a diverse array of substrates, spanning from the synthesis of simple catechols to intricate phenylpropanoids and isoquinoline alkaloids [35, 36]. Previous studies revealed *IiOMT3* methylated the 3'-hydroxy moiety of flavonoids such as eriodictyol and 3'-hydroxydaizein. Additionally, it methylated the 7-OH positions of flavones and facilitated the conversion of caffeic acid into ferulic acid [37]. In this study, substrate binding and specificity in plant *O*-methyltransferases were verified by the results of widely targeted metabolomics of *P. cyrtonema* and overexpressed transgenic *N. benthaniana*. Meanwhile, the four genes (*PcOMT10/11/12/13*) mined in *P. cyrtonema* showed similar function to those in *N. benthaniana* for catalyzing the generation of target products from substrates, demonstrating that these enzymes are relatively functionally conserved for the catalytic mechanism among different species. Among them, the *PcOMT12* gene played a positive role in different target compound accumulation in multiple reactions and significantly increased chrysoeriol-6,8-di-C-glucoside content after overexpression in *N. benthaniana*, which is presumed to have high catalytic activity and widely catalytic substrates. It is expected to be further exploited in the future.

In this study, we used machine learning for the first time to identify three significant metabolite markers between Qingyuan and Xiushui counties in *P. cyrtonema.* Then multi-omics approach were used to mine and verify candidate genes (*PcOMT10/11/12/13*) with over-expressed transient expression in *N. benthaniana* leaves. These results offer novel insights into the molecular basis of phenylpropanoid accumulation in *P. cyrtonema*.

## Conclusion

*Polygonatum cyrtonema* Hua is an ancient and traditional medicinal plant in China. This study focused on the metabolomics of *P. cyrtonema* growing in Qingyuan County (Zhejiang Province) and Xiushui County (Jiangxi Province). Based on machine learning, we discovered three metabolite markers quercetin-3-O-(6"-malonyl) galactoside, epipinoresinol, salicin. Additionally, comparative transcriptome analysis mined four *O*-methyltransferases *PcOMT10/11/12/13*. Additionally, WGCNA helped to identify potential candidate genes associated with flavonoids such as *CHI*, *UGT1*, *UGT2*, ERF, as well as the phenolic acids-related genes *UGT3* and *NAC*. Furthermore, transient overexpression of four OMTs were conducted in *N. benthaniana* leaves validating the role in altering metabolic flow for accumulating phenylpropanoids. These results are expected to provide an important basis for metabolites substance and subsequent genetic studies applied for precision breeding of *P. cyrtonema* facilitating the production of high-value-added medicinal products.

### Supplementary Information

**Supplementary Material 1.**

**Supplementary Material 2.**

## Data Availability

Transcriptome data can be found in NCBI (https://www.ncbi.nlm.nih.gov/), and the submission numbers is SUB13866596.
