# Supplementary material for: Accumulation mechanism of metabolite markers identified by machine learning between Qingyuan and Xiushui counties in Polygonatum cyrtonema Hua
Source: BMC Plant Biol. 2024 Mar 6;24:173. doi: 10.1186/s12870-024-04871-6 (PMC10916035; doi:10.1186/s12870-024-04871-6)
Supplement: Supplementary file 2 — Supplementary Material 2. [file 12870_2024_4871_MOESM2_ESM.pptx]

## Slide 1
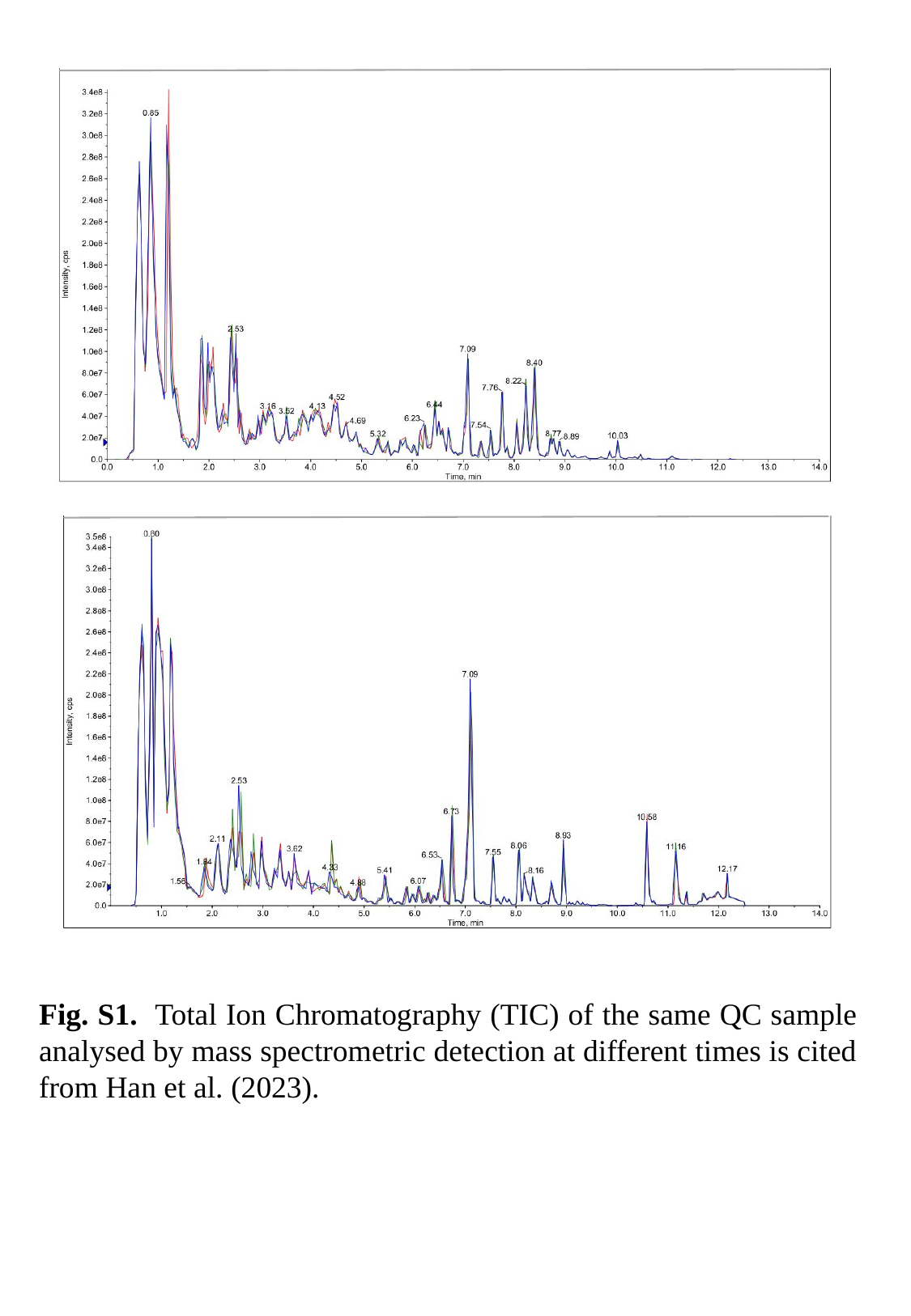

Fig. S1. Total Ion Chromatography (TIC) of the same QC sample analysed by mass spectrometric detection at different times is cited from Han et al. (2023).

## Slide 2
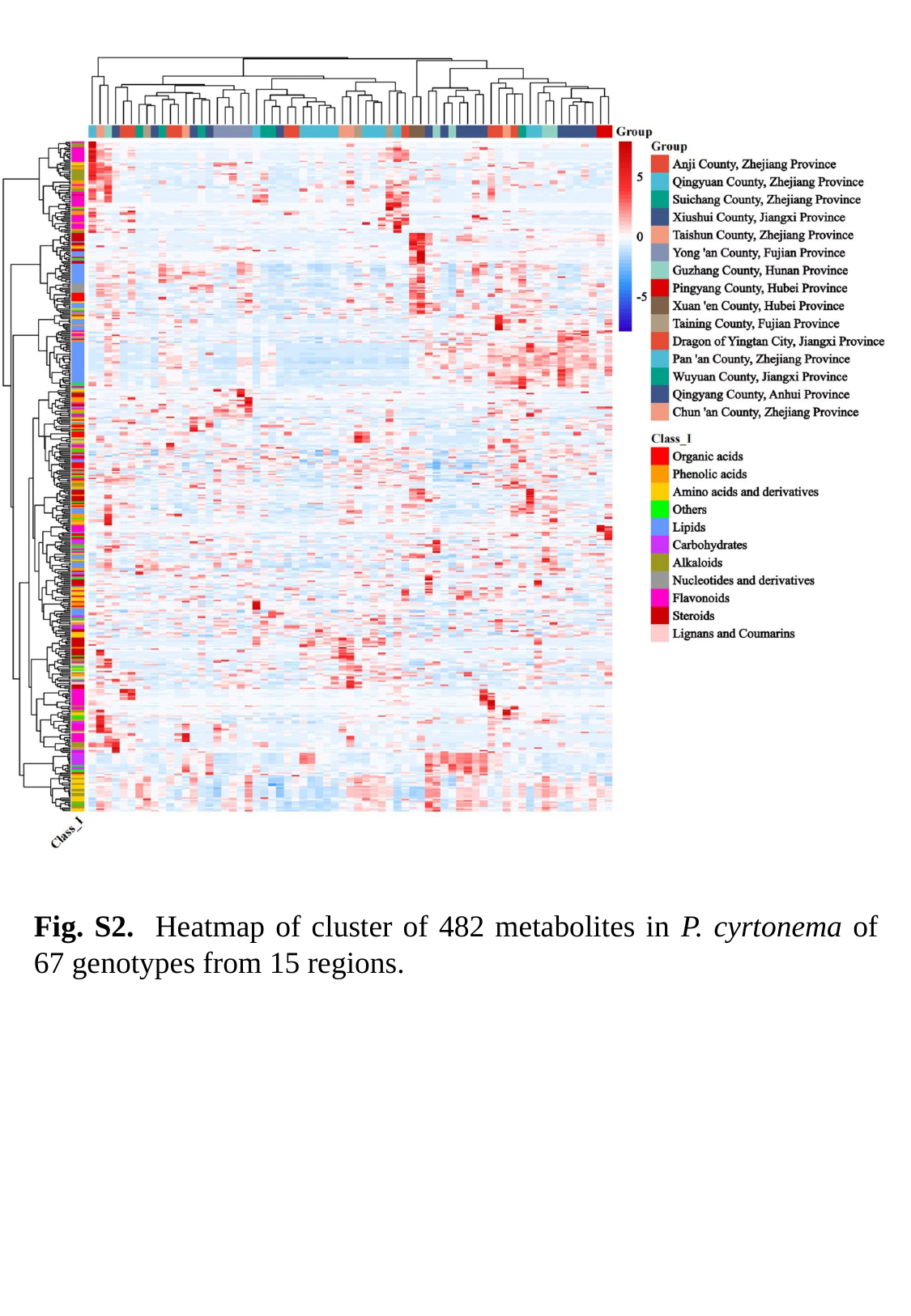

Fig. S2. Heatmap of cluster of 482 metabolites in P. cyrtonema of 67 genotypes from 15 regions.

## Slide 3
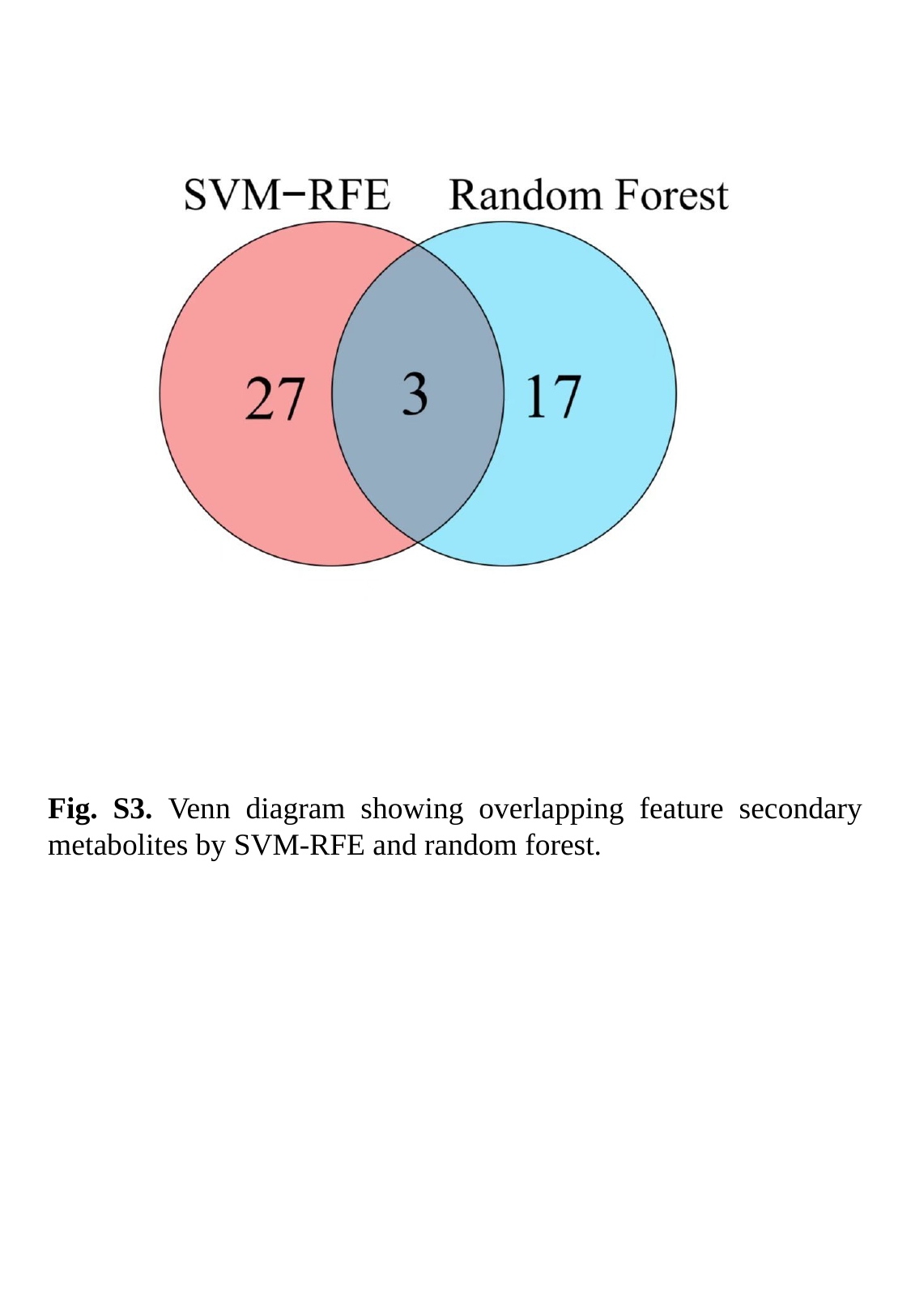

Fig. S3. Venn diagram showing overlapping feature secondary metabolites by SVM-RFE and random forest.

## Slide 4
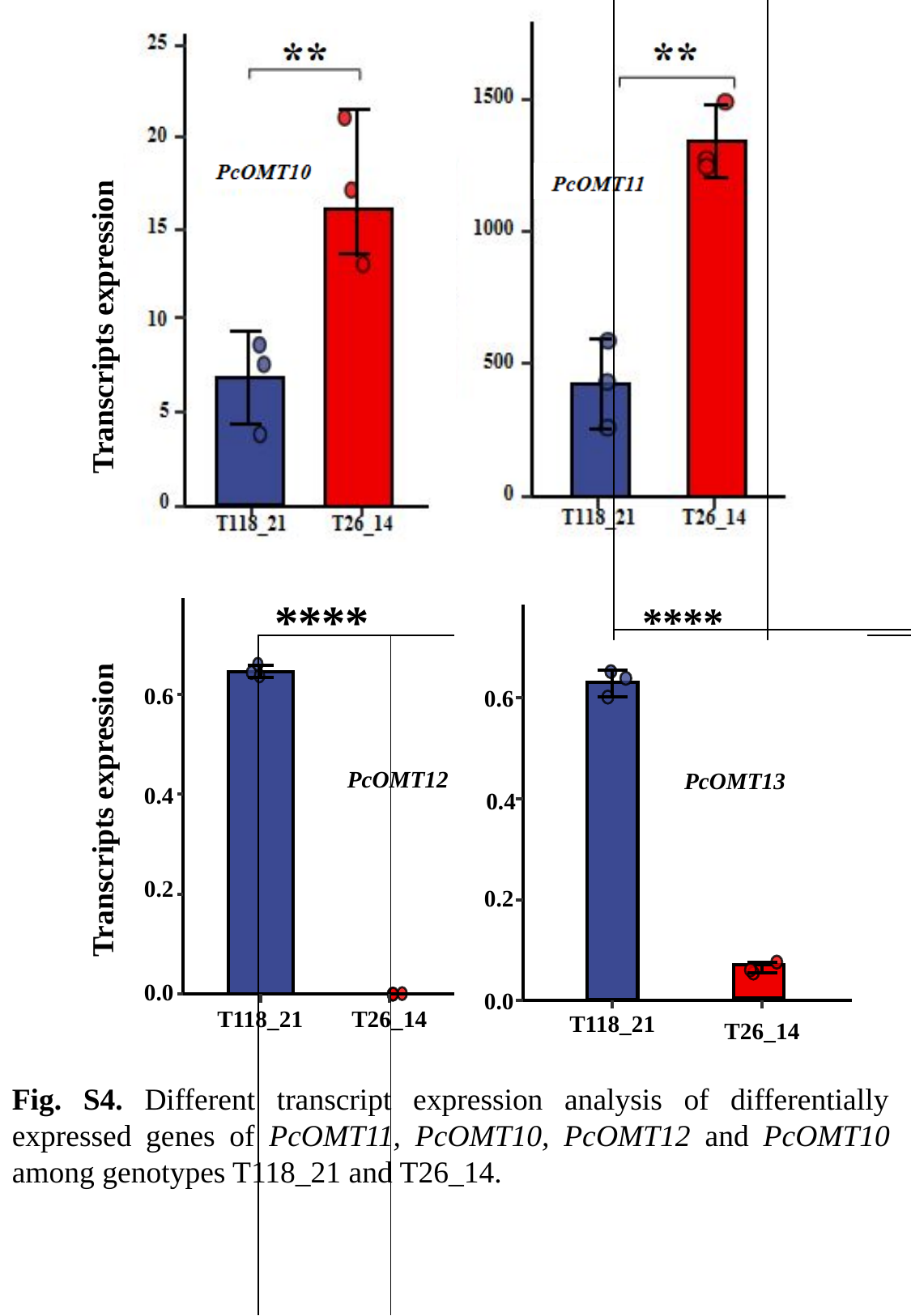

Transcripts expression
****
0.6
0.4
0.2
0.0
T118_21
T26_14
****
0.6
0.4
0.2
0.0
T118_21
T26_14
PcOMT13
Transcripts expression
PcOMT12
Fig. S4. Different transcript expression analysis of differentially expressed genes of PcOMT11, PcOMT10, PcOMT12 and PcOMT10 among genotypes T118_21 and T26_14.

## Slide 5
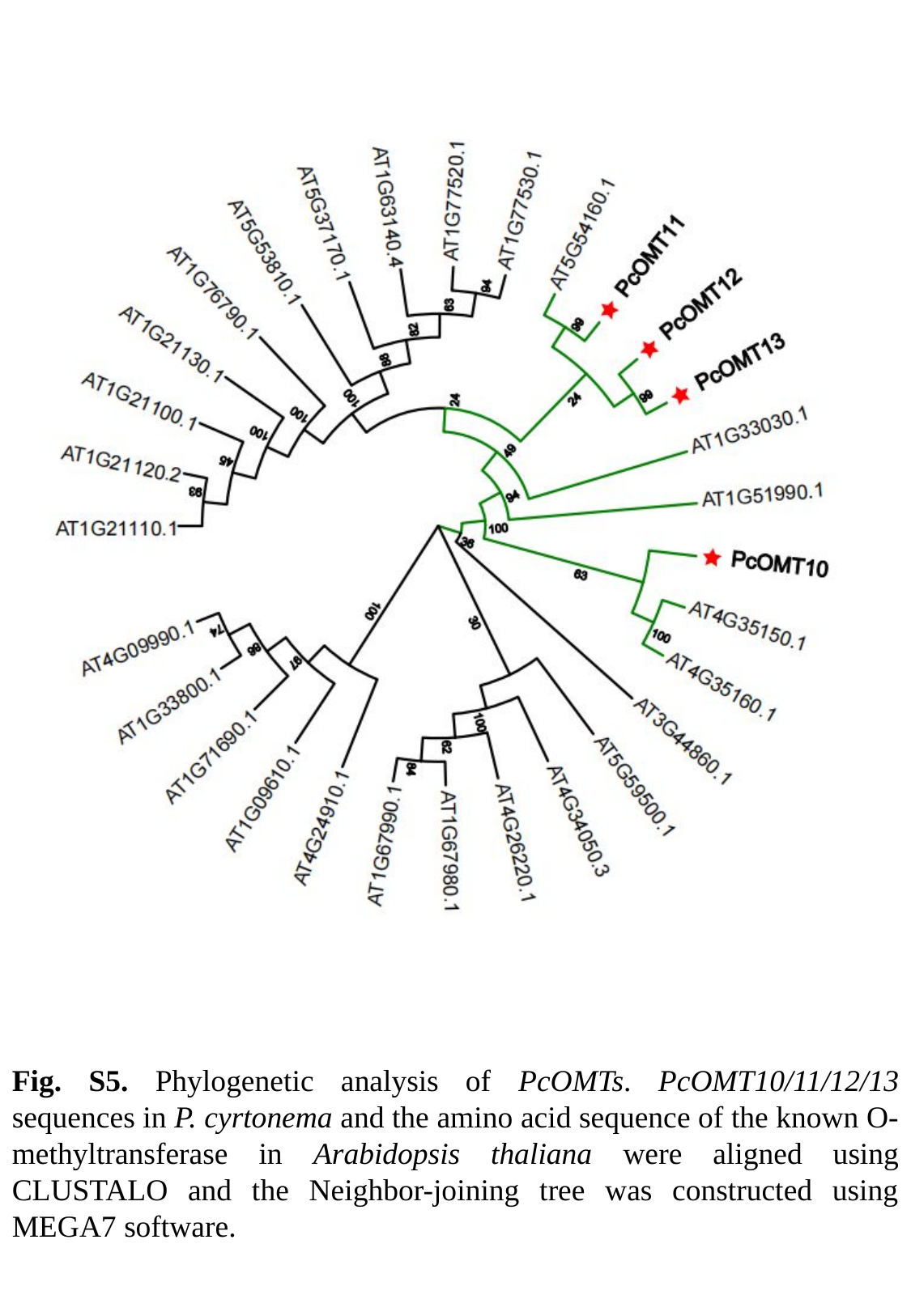

Fig. S5. Phylogenetic analysis of PcOMTs. PcOMT10/11/12/13 sequences in P. cyrtonema and the amino acid sequence of the known O-methyltransferase in Arabidopsis thaliana were aligned using CLUSTALO and the Neighbor-joining tree was constructed using MEGA7 software.

## Slide 6
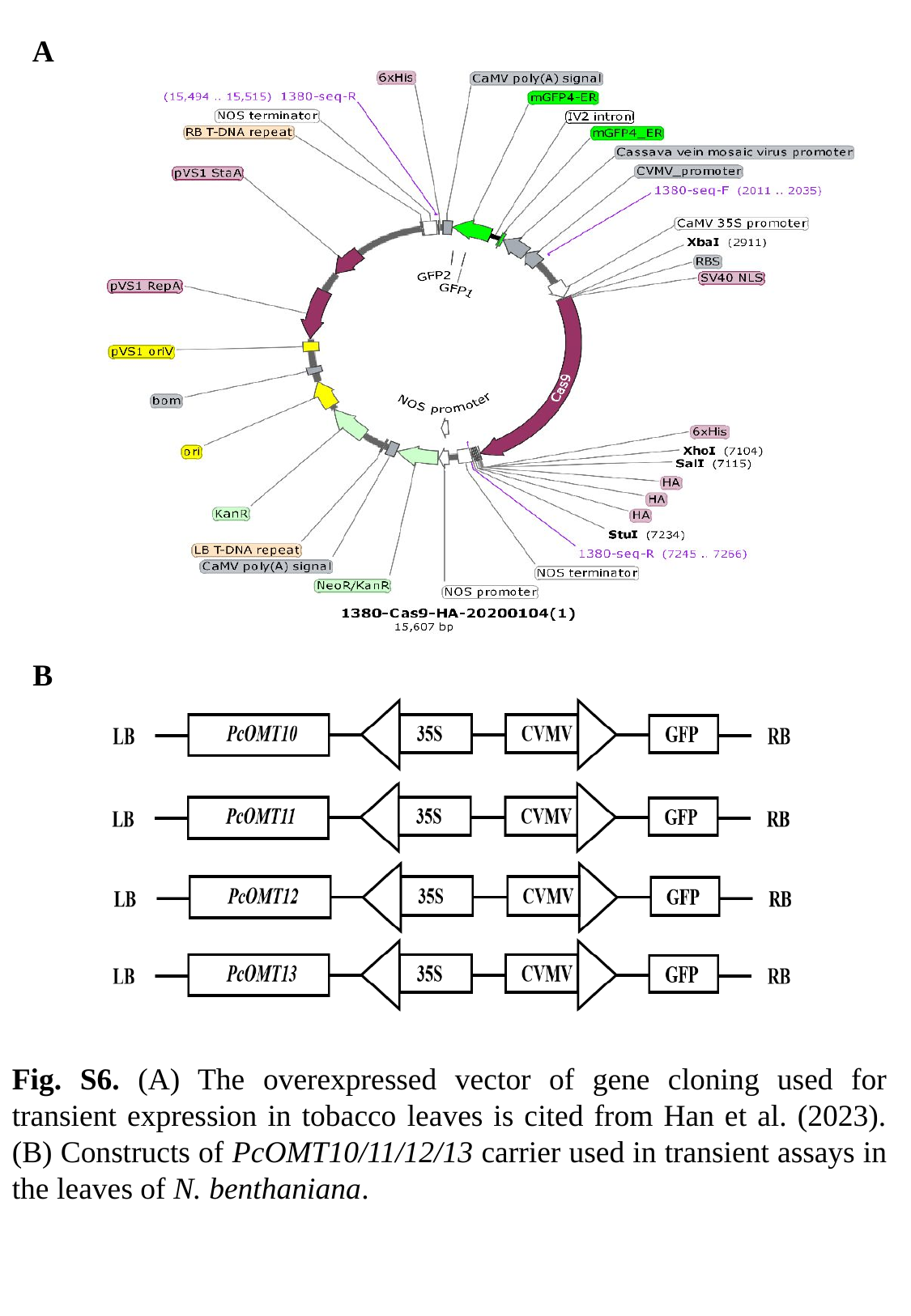

A
B
Fig. S6. (A) The overexpressed vector of gene cloning used for transient expression in tobacco leaves is cited from Han et al. (2023). (B) Constructs of PcOMT10/11/12/13 carrier used in transient assays in the leaves of N. benthaniana.

## Slide 7
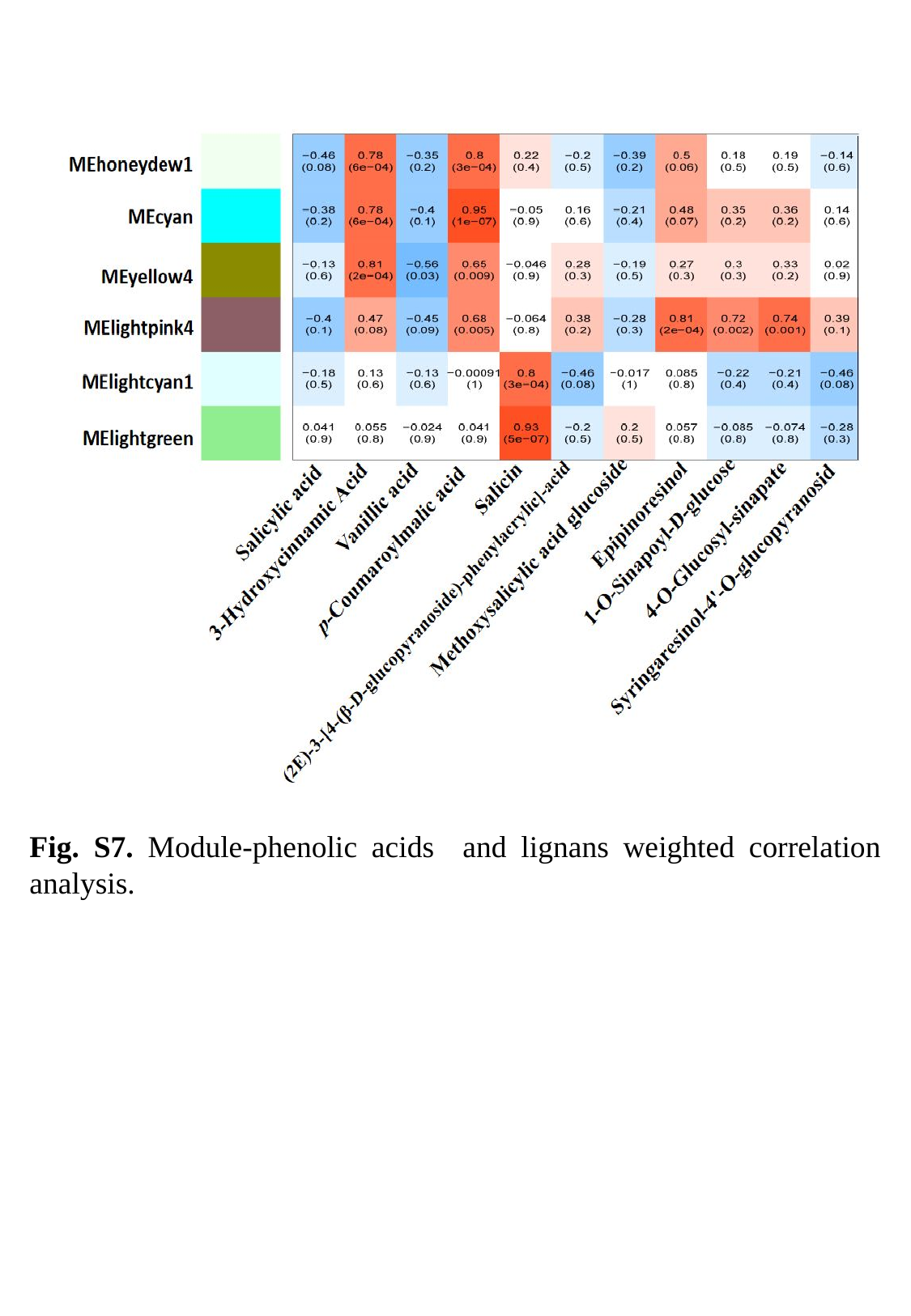

Fig. S7. Module-phenolic acids and lignans weighted correlation analysis.
